# Recent advances in understanding of the epigenetic regulation of plant regeneration

**DOI:** 10.1007/s42994-022-00093-2

**Published:** 2023-01-16

**Authors:** Xuemei Liu, Kehui Zhu, Jun Xiao

**Affiliations:** 1 Key Laboratory of Plant Cell and Chromosome Engineering, Institute of Genetics and Developmental Biology, Chinese Academy of Sciences, Beijing, 100101 China; 2 University of Chinese Academy of Sciences, Beijing, 100049 China; 3 CAS-JIC Centre of Excellence for Plant and Microbial Science (CEPAMS), Institute of Genetics and Developmental Biology, Chinese Academy of Sciences, Beijing, 100101 China

**Keywords:** Plant regeneration, Epigenetic regulation, *Arabidopsis*, Crop breeding

## Abstract

Ever since the concept of “plant cell totipotency” was first proposed in the early twentieth century, plant regeneration has been a major focus of study. Regeneration-mediated organogenesis and genetic transformation are important topics in both basic research and modern agriculture. Recent studies in the model plant *Arabidopsis thaliana* and other species have expanded our understanding of the molecular regulation of plant regeneration. The hierarchy of transcriptional regulation driven by phytohormone signaling during regeneration is associated with changes in chromatin dynamics and DNA methylation. Here, we summarize how various aspects of epigenetic regulation, including histone modifications and variants, chromatin accessibility dynamics, DNA methylation, and microRNAs, modulate plant regeneration. As the mechanisms of epigenetic regulation are conserved in many plants, research in this field has potential applications in boosting crop breeding, especially if coupled with emerging single-cell omics technologies.

## Introduction

From unicellular green algae to angiosperms, plants are highly regenerative, meaning that new organs or whole bodies can be rebuilt following injury (Ikeuchi et al. 2016, 2019). Research on regeneration can be traced back to Gottlieb Haberlandt’s description of totipotency proposed in the early twentieth century (Haberlandt 2003; Krikorian and Berquam 2003; Thorpe 2007). In 1957, Skoog and Miller demonstrated that the ratio of exogenous auxin to cytokinin (CK) affects the fate of callus differentiation in tobacco (*Nicotiana tabacum*) (Skoog and Miller 1957), providing experimental tools and a conceptual framework for exploring the functions of phytohormones and their interactions during regeneration (Birnbaum and Alvarado 2008). Plant tissue culture has been widely used in both basic research and agriculture and provides an excellent system for studying plant organogenesis and somatic embryogenesis. Strategies that combine tissue culture and genome editing technologies provide opportunities to genetically improve crops (Loyola-Vargas and Ochoa-Alejo 2018).

Plant regeneration can be divided into two categories: injury-induced regeneration and tissue culture-induced regeneration (Mathew and Prasad 2021). In the former, different regeneration processes occur depending on the type of injury (Ikeuchi et al. 2016). When the meristem is partially damaged, the plant will reconstruct the meristem, whereas when the meristem is completely absent, the plant will grow axillary shoots or lateral roots. Some plant species, such as those in the *Crassulaceae* and *Gesneriaceae* families, can undergo de novo organogenesis to form new shoots or roots from cut sites (Ikeuchi et al. 2016). Other plants, however, require tissue culture to regenerate the entire plant. Injury activates a range of genes, including cell cycle genes, genes involved in CK synthesis and responses, and genes encoding transcription factors (TFs) of the AP2/ERF family (Ikeuchi et al. 2017). *WOUND INDUCED DEDIFFERENTIATION 1* (*WIND1*) is rapidly induced at the wounding site and promotes cell dedifferentiation to form callus via type-B ARR (Iwase et al. 2011). Another gene in the AP2/ERF family, *ETHYLENE RESPONSE FACTOR 115* (*ERF115*), promotes reconstitution of the stem cell niche after root tip excision (Heyman et al. 2016).

Tissue culture-induced regeneration can be divided into three types based on the culture system and regeneration process used: de novo root regeneration, de novo shoot regeneration, and somatic embryogenesis (Mathew and Prasad 2021). Both de novo root regeneration and shoot regeneration are two-step processes. The explant forms callus on an auxin-rich callus induction medium (CIM). The callus then differentiates into roots on root induction medium (RIM), which contains little or no auxin, or shoots on CK-rich shoot induction medium (SIM) (Lardon and Geelen 2020). Regardless of the origin of the explant, the process of callus formation induced on CIM follows the root developmental pathway (Sugimoto et al. 2010), and the identity of the callus is similar to that of root primordia (Zhai and Xu 2021). During this process, auxin signaling in *Arabidopsis* (*Arabidopsis thaliana*) first activates *WUSCHEL RELATED HOMEOBOX 11* (*WOX11*) and *WOX12*, which transforms the regenerative pericycle or pericycle-like cells of the explant into root founder cells (Atta et al. 2009; Liu et al. 2014; Sang et al. 2018; Xu 2018). Subsequently, in the continuous presence of auxin, WOX11/WOX12 activates the expression of *WOX5*, *WOX7*, *LATERAL ORGAN BOUNDARIES-DOMAIN 16* (*LBD16*), and *LBD29*, which in turn transform root founder cells into root primordium cells (Hu and Xu 2016; Xu 2018; Liu et al. 2018b). Thus, callus is formed on CIM.

Subsequently, the ratio of auxin to CK determines the direction of callus re-differentiation. On RIM, callus resembling root primordia continues to undergo cell division and differentiates into a well-organized root apical meristem (RAM). During this process, *LBD16* expression in the root meristem gradually decreases and the expression of *WOX5* and *WOX7* is restricted to the stem cell niche (Hu and Xu 2016; Xu 2018; Jing et al. 2020). In addition, *PLETHORA 1* (*PLT1*), *PLT2*, *SCARECROW* (*SCR*), and *SHORT ROOT* (*SHR*) are essential for quiescent center specification and stem cell activity in the RAM (Della Rovere et al. 2015; Shimotohno et al. 2018). However, under the induction of CK in SIM, the callus differentiates into shoots. First, the expression of *CUP-SHAPED COTYLEDON1* (*CUC1*) and *CUC2* in callus is spatially reorganized to mark promeristem regions, and *PIN-FORMED 1* (*PIN1*) is induced by CUCs to determine the future locations of shoot progenitors (Hibara et al. 2003; Daimon et al. 2003; Gordon et al. 2007; Shin et al. 2020). Along with the up-regulation of *PIN1*, *SHOOT MERISTEMLESS* (*STM*) is expressed in the promeristem to maintain shoot meristem activity (Gordon et al. 2007; Shin et al. 2020). In addition, type-B ARRs in the CK signaling pathway, including ARABIDOPSIS RESPONSE REGULATOR 1 (ARR1), ARR2, ARR10, and ARR12, directly bind to and activate *WUSCHEL* (*WUS*), which directs the shoot apical meristem (SAM) formation program (Negin et al. 2017; Zhang et al. 2017b; Shin et al. 2020).

Unlike de novo root or shoot regeneration, somatic embryogenesis leads to the formation of a bipolar structure with an apical and basal pole. In *Arabidopsis*, somatic embryogenesis is often induced from 2,4-dichlorophenoxyacetic acid (2,4-D)-treated immature zygotic embryos at the bent cotyledon stage of development (Horstman et al. 2017). Ectopic expression of the embryo identity genes *LEAFY COTYLEDON 1* (*LEC1*) (Lotan et al. 1998) and *LEC2* (Stone et al. 2001), the meristem identity genes *BABY BOOM* (*BBM*) (Boutilier et al. 2002) and *WUS* (Gaj 2004; Chatfield et al. 2013), and wound-induced *WIND1* (Ikeuchi et al. 2013) induces somatic embryogenesis.

**Fig. 1:**
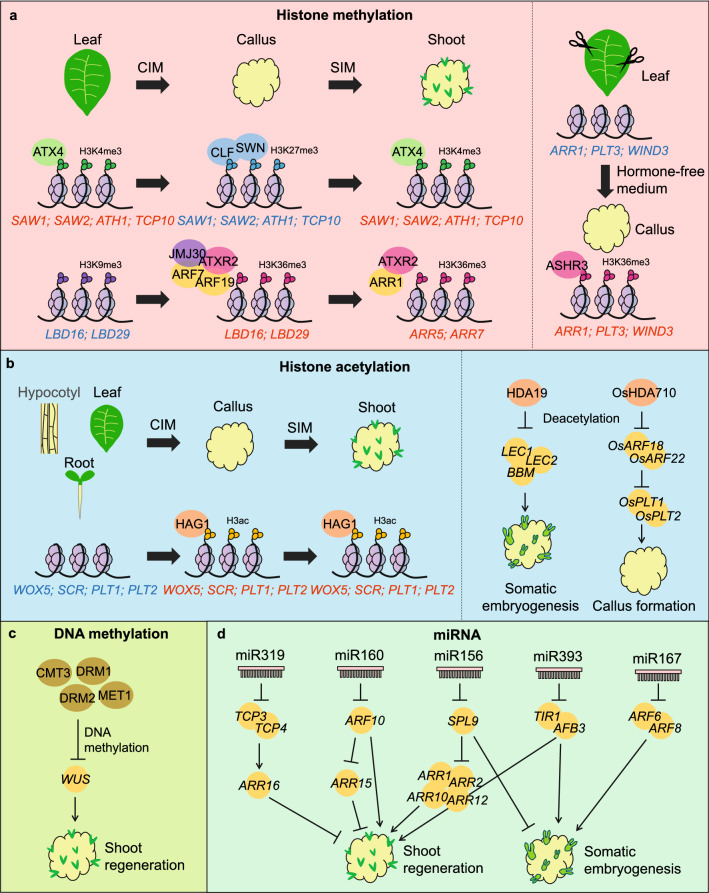
Roles of epigenetic regulators in plant regeneration. Mechanisms of histone methylation (**A**), histone acetylation (**B**), DNA methylation (**C**)**,** and miRNA (**D**) in regulating plant regeneration. The font color indicates the transcriptional status of the gene, with red representing transcriptional activation and blue representing transcriptional repression. The scissors represent the injury-induced regeneration. The arrows represent activation and the T-ended arrows represent inhibition

Plant cells undergo multiple rapid cell fate transitions during regeneration, which is accompanied by the reprogramming of the transcriptome and chromatin landscape. Cell identity genes, especially TF genes, are induced by phytohormones to participate in plant regeneration (Sang et al. 2018; Ikeuchi et al. 2019; Sugimoto et al. 2019; Mathew and Prasad 2021). The expression of these key TF genes is partially regulated by various epigenetic regulators. In 2007, Crane and Gelvin reported that RNAi lines in which 24 genes encoding epigenetic regulators, including chromatin remodeling complexes, DNA methyltransferases, and various histone modification-related enzymes, had been silenced showed altered genetic transformation efficiencies (Crane and Gelvin 2007). Further studies have uncovered epigenetic dynamics during plant regeneration and highlighted the importance of the epigenetic regulation of key TFs that drive the cell fate transition during regeneration (Wang et al. 2020; Xu et al. 2021; Wu et al. 2022). Here, we summarize recent advances in understanding the epigenetic regulation of the plant regeneration process (Fig. 1, Table 1), with a focus on tissue culture-induced regeneration, and propose future directions for better understanding the different layers of epigenetic regulation of plant regeneration and their applications in crop breeding.

**Table 1:** Epigenetic regulators of plant regeneration

Protein name	Protein ID	Annotation	Targets	Roles in regeneration	References
CLF	AT2G23380	H3K27me3 methyltransferase	*SAW1*, *SAW2*, *ATH1*, *TCP10*	Inhibits somatic embryogenesis; Promotes callus formation from leaves	He et al. (2012)
SWN	AT4G02020	H3K27me3 methyltransferase	*SAW1*, *SAW2*, *ATH1*, *TCP10*	Inhibits somatic embryogenesis; Promotes callus formation from leaves	He et al. (2012)
ATX4	AT4G27910	H3K4me3 methyltransferase	*ATH1*, *KANT4*, *SAW1*, *SAW2*, *TCP10*, *YAB5*	Inhibits callus formation from leaves; Promotes shoot regeneration from callus	Lee et al. (2019)
LDL3	AT4G16310	H3K4me2 demethylase	*CIPK23*, *GLT1, UPL4*, *ARR12*	Promotes shoot regeneration from callus	Ishihara et al. (2019)
ATXR2	AT3G21820	H3K36me3 methyltransferase	*LBD16*, *LBD29*, *ARR5*, *ARR7*	Promotes callus formation from leaves; Inhibits shoot regeneration from callus	Lee et al. (2017, 2018b, 2021)
ASHR3	AT4G30860	H3K36me3 methyltransferase	*ARR1*, *PLT3*, *WIND3*	Promotes wound-induced callus formation	Lee et al. (2020)
JMJ30	AT3G20810	H3K9me3 demethylase	*LBD16*, *LBD29*	Promote callus formation from leaves	Lee et al. (2018a)
AtPRMT5	AT4G31120	H4R3sme2 methyltransferase	*KRP1*	Promotes shoot regeneration from callus	Liu et al. (2016a)
HAG1	AT3G54610	histone acetyltransferase	*WOX5*, *SCR*, *PLT1*, *PLT2*	Promotes wound-induced callus formation; Promotes shoot regeneration from callus	Kim et al. (2018), Rymen et al. (2019)
HAG3	AT5G50320	histone acetyltransferase	*NA*	Promotes wound-induced callus formation	Rymen et al. (2019)
HDA19	AT4G38130	histone deacetylases	*LEC2*	Inhibits somatic embryogenesis	Morończyk et al. (2022)
HDA9	AT3G44680	histone deacetylases	*LBD17, LEC1*	Inhibits callus formation from leaves	(Lee et al. 2016)
HDT1	AT3G44750	histone deacetylases	*BBM, WUS*	Inhibits callus formation from leaves	Lee et al. (2016)
OsHDA710	Os02g0215200	histone deacetylases	*OsARF18, OsARF22*	Promote callus formation from embryos	Zhang et al. (2020)
HTR15	AT5G12910	coding H3.15	*WOX11*	Promotes wound-induced callus formation; Promotes callus formation from hypocotyls	Yan et al. (2020)
INO80	AT3G57300	chromatin remodeling complexes	*PIN1*	Collaborates with histone chaperones NRP1/2 to regulate IM and RAM activities	Kang et al. (2019)
PKL	AT2G25170	chromatin remodeling complexes	*LEC1*, *FUS3*, *ABI3*, *EMF2*, *CLF, SWN, AP3, AG, FLC*	Facilitates root meristem activity; Limits embryogenesis	Aichinger et al. (2009, 2011)
BRM	AT2G46020	chromatin remodeling complexes	*Cyc81;1, CycB1;3*, *PIN1–4*, *PIN7*	Maintains the root stem cell niche	Yang et al. (2015)
CHR3	AT2G28290	chromatin remodeling complexes	*WUS*	Maintains the floral meristem	Sun et al. (2019)
MET1	AT5G49160	DNA methyltransferase	*WUS*, *ARR1*, *ARR10*, *CRY1*	Inhibits shoot regeneration from callus	Liu et al. (2018a), Shim et al. (2021)
DRM1	AT5G15380	DNA methyltransferase	*WUS*	Inhibits shoot regeneration from callus	Shemer et al. (2015)
DRM2	AT5G14620	DNA methyltransferase	*WUS*	Inhibits shoot regeneration from callus	Shemer et al. (2015)
CMT3	AT1G69770	DNA methyltransferase	*WUS*	Inhibits shoot regeneration from callus	Shemer et al. (2015)
miR156		microRNA	*SPL9*	Promotes shoot regeneration; Promotes somatic embryogenesis	Zhang et al. (2015), Long et al. (2018)
miR319		microRNA	*TCP3*, *TCP4*	Inhibits shoot regeneration	Yang et al. (2020)
miR160		microRNA	*ARF10*	Inhibits callus formation; Inhibits shoot regeneration	Qiao et al. (2012), Liu et al. (2016b)
miR167		microRNA	*ARF6*, *ARF8*	Inhibits somatic embryogenesis	Arora et al. (2020)
miR393		microRNA	*TIR1*, *AFB3*	Inhibits shoot regeneration; Inhibits somatic embryogenesis	Wójcik and Gaj (2016), Wang et al. (2018)

## Layers of epigenetic regulation of transcription

Gene transcription is regulated at multiple levels. In general, trans-acting TFs bind to cis-elements in their target promoters to promote or inhibit gene transcription. However, in the cellular environment, DNA is wrapped around histones and packaged into nucleosomes, which limits the access of TFs. Transcription factors compete with histones and other chromatin-binding proteins to bind to DNA (Klemm et al. 2019). Chromatin remodeling complexes can directly alter nucleosome composition and interactions, thereby affecting chromatin accessibility (Ojolo et al. 2018). Methylation or acetylation of histone H3 and H4 can affect the interactions between histones and DNA, resulting in loose or dense chromatin (Pfluger and Wagner 2007). DNA methylation is also involved in transcriptional regulation. DNA methylation in promoters inhibits gene transcription, whereas DNA methylation in gene bodies is mostly associated with constitutive expression (Zhang et al. 2018a). Post-transcriptional regulation, such as processes mediated by microRNA (miRNA), can also affect gene expression (Gibney and Nolan 2010).

## Regulation of plant regeneration via histone modifications and histone variants

### Histone methylation-regulated plant regeneration

The lysine or arginine residues of histone tails can be modified by mono-, di- and tri-methylation, which affects transcription by altering the local chromatin state. Methylation of residues at different positions of histone has various effects on the transcriptional regulation of genes. In general, trimethylation at lysine 27 of histone H3 (H3K27me3) and trimethylation at lysine 9 of histone H3 (H3K9me3) negatively regulate transcription, whereas trimethylation at lysine 4 of histone H3 (H3K4me3) and trimethylation at lysine 36 of histone H3 (H3K36me3) are associated with transcriptional activation (Xiao et al. 2016). Histone methylation dynamics are regulated by “writers” that add methyl groups, such as histone lysine methyltransferase (HKMTs) and protein arginine methyltransferases (PRMTs), and “erasers” that remove methyl group, such as histone demethylases (HDMs) (Liu et al. 2010).

#### H3K27me3 dynamics regulate plant regeneration

In *Arabidopsis*, H3K27me3 is catalyzed by Polycomb repressive complex 2 (PRC2), which consists of four subunits. The catalytic subunit Enhancer of zeste homolog2 (EZH2) is encoded by three functionally independent genes: *CURLY LEAF* (*CLF*), *SWINGER* (*SWN*), and *MEDEA* (*MEA*). The spatial–temporal-specific expression of these genes leads to the functional diversification of PRC2 (Bieluszewski et al. 2021). Instead of exhibiting normal plant architecture, the *clf swn* double mutant spontaneously forms a callus-like tissue that accumulates neutral lipids and occasionally somatic embryo-like structures (Chanvivattana et al. 2004; Ikeuchi et al. 2015). Although auxin failed to induce somatic embryogenesis from wild-type shoots, this treatment induced somatic embryo formation from *clf swn* shoots (Mozgová et al. 2017). This observation suggests that the loss of function of PRC2 promotes somatic embryogenesis from vegetative tissues in *Arabidopsis*. In rice (*Oryza sativa*), the H3K27me3 levels of *OsWOX6*, *OsWOX9*, *OsWOX11*, *OsPLT3*, *OsPLT6*, and *OsPLT8* are significantly lower in callus than in seedlings (Zhao et al. 2020). In peach (*Prunus persica*), during the leaf-to-callus transition, the up-regulation of auxin-related genes *PpPIN6* and *AUXIN-INDUCED PROTEIN 13* (*PpIAA13*) and the lateral root development-related genes *PpLBD1*, *LATERAL ORGAN BOUNDARIES* (*PpLOB*), *SHI RELATED SEQUENCE 1* (*PpSRS1*), and *LATERAL ROOT PRIMORDIUM 1* (*PpLRP1*) is accompanied by a decrease in H3K27me3 levels (Zheng et al. 2022). Furthermore, treatment with the H3K27me3 demethylase inhibitor GSK-J4 significantly reduced the rate of callus induction in peach (Zheng et al. 2022). In hexaploid wheat (*Triticum aestivum*), H3K27me3 deceases at auxin signaling genes and root meristem formation-related genes such as *TaPIN1* and *TaLBD17* during the late stage of callus formation from immature embryos (Liu et al. 2022b). Thus, the attenuation of H3K27me3 of auxin signaling and meristematic-related genes could facilitate their activation and promote callus formation. By contrast, leaf tissue of the *Arabidopsis clf swn* mutant failed to form callus on CIM, because leaf identity genes, such as *SAWTOOTH 1* (*SAW1*), *SAW2*, *ARABIDOPSIS THALIANA HOMEOBOX GENE 1* (*ATH1*), and *TCP DOMAIN PROTEIN 10* (*TCP10*), cannot be repressed without PRC2-mediated H3K27me3 deposition during dedifferentiation and callus induction (Fig. 1A) (He et al. 2012). In summary, PRC2-mediated H3K27me3 marks meristematic genes in differentiated tissues to prevent callus formation, while it is also required to turn off tissue identity genes for the acquisition of pluripotency and to facilitate callus formation.

#### Altering H3K4me3 and H3K4me2 affects plant regeneration

During the leaf-to-callus transition in *Arabidopsis*, the down-regulation of leaf identity genes is also regulated by the decrease in H3K4me3 levels. ARABIDOPSIS TRITHORAX 4 (ATX4) (Foroozani et al. 2021) catalyzes the trimethylation of H3K4 and participates in de novo shoot regeneration from leaf explants (Lee et al. 2019). ATX4 is highly expressed in leaves and deposits H3K4me3 on the leaf identity genes *ATH1*, *KNOTTED1-LIKE HOMEOBOX GENE 4* (*KNAT4*), *SAW1*, *SAW2*, *TCP10*, and *YABBY 5* (*YAB5*) to maintain leaf cell identify. Upon induction on CIM, the expression level of *ATX4* decreases rapidly and remains low throughout callus induction. As a result, the H3K4me3 and expression levels of leaf identity genes decrease, which results in the loss of leaf cell identity (Lee et al. 2019). Compared to the wild type, *atx4* more readily generates callus from leaf tissue (Lee et al. 2019). *ATX4* also affects re-differentiation from callus to shoot tissue. When callus is transferred to SIM, *ATX4* is temporarily up-regulated, and ATX4 re-deposits H3K4me3 on *ATH1*, *SAW1*, *SAW2*, *TCP10*, and *YAB5* to regain shoot identity (Fig. 1A) (Lee et al. 2019). Thus, ATX4-mediated H3K4me3 has dual functions in callus induction and the re-differentiation of callus in *Arabidopsis*. By contrast, in crops such as wheat (Liu et al. 2022b) and rice (Zhao et al. 2020), H3K4me3 deposition increases during immature embryo- or seed-induced callus formation, pointing to an opposite role for H3K4me3 in promoting callus formation in these crops compared to *Arabidopsis*. This discrepancy might be due to the different types of explants used for each species.

In general, dimethylation at lysine 4 of histone H3 (H3K4me2) positively regulates gene expression in animals (Barski et al. 2007) but is associated with gene repression in plants (Liu et al. 2019). LYSINE-SPECIFIC DEMETHYLASE 1-LIKE 3 (LDL3) accumulates and specifically erases H3K4me2 marks on genes required for the acquisition of shoot traits during callus formation from root cells (Ishihara et al. 2019). Interestingly, LDL3-mediated removal of H3K4me2 does not immediately activate target genes, but rather primes genes for subsequent activation during shoot induction on SIM (Ishihara et al. 2019). Accordingly, the competency of shoot regeneration is severely impaired in the *ldl3* mutant due to its failure to eliminate H3K4me2 on shoot regeneration genes, such as *CBL-INTERACTING PROTEIN KINASE 23* (*CIPK23*) and *UBIQUITIN-PROTEIN LIGASE 4* (*UPL4*) (Ishihara et al. 2019).

#### The status of H3K36me3 and H3K9me3 influences plant regeneration

Signaling factors in the auxin and CK pathways interact with histone modifiers to regulate gene expression during plant regeneration. The H3K36me3 methyltransferase gene *ARABIDOPSIS TRITHORAX-RELATED 2* (*ATXR2*) (Lee et al. 2017) and the H3K9me3 demethylase gene *JUMONJI C DOMAIN-CONTAINING PROTEIN 30* (*JMJ30*) (Lee et al. 2018a) are constitutively up-regulated upon callus induction on CIM. The auxin signaling factors AUXIN RESPONSE FACTOR 7 (ARF7) and ARF19 interact with and recruit ATXR2 and JMJ30 to the promoters of *LBD16* and *LBD29* and activate their expression to promote the dedifferentiation of leaf explants and callus formation (Fig. 1A) (Lee et al. 2017, 2018a). Notably, both JMJ30-mediated decreases in H3K9me3 and ATXR2-mediated increases in H3K36me3 are required for the activation of *LBDs* during the leaf-to-callus transition. ATXR2 also promotes root organogenesis on a phytohormone-free medium (Lee et al. 2018b) and inhibits shoot regeneration on SIM (Lee et al. 2021). The *atxr2* mutant shows enhanced shoot regeneration from the callus regardless of the origin of the explant (Lee et al. 2021). ATXR2 interacts with type-B ARR1 in the CK signaling pathway to deposit H3K36me3 and activate *ARR5* and *ARR7* on SIM. ARR5 and ARR7 are type-A ARRs that inhibit *WUS* expression and shoot formation (Lee et al. 2021). Therefore, auxin-inducible ATXR2 regulates CK signaling and precisely controls *WUS* expression to prevent premature shoot induction. Another H3K36me3 methyltransferase, ASH1-RELATED 3 (ASHR3), promotes wound-induced callus formation. Following wounding, ASHR3 is rapidly activated and deposits H3K36me3 on *ARR1*, *PLT3*, and *WIND3* to promote callus formation (Fig. 1A) (Lee et al. 2020).

#### Role of H4R3sme2 in plant regeneration

In addition to lysine methylation, histone arginine methylation is also involved in plant regeneration. *Arabidopsis* PROTEIN ARGININE METHYLTRANSFERASE 5 (PRMT5), which catalyzes the symmetric dimethylation at arginine 3 of histone H4 (H4R3sme2) and RNA splicing factors, affects the transcription and protein levels of the cyclin-dependent kinase inhibitor KIP-RELATED PROTEIN 1 (KRP1) to participate in shoot regeneration (Liu et al. 2016a). PRMT5 deposits H4R3sme2 on *KRP1* and *KRP2* to inhibit their transcription (Liu et al. 2016a). Furthermore, AtPRMT5 affects the alternative splicing of the E3 ubiquitin ligase gene *RELATED TO KPC1* (*RKP*), which produces an abnormal RKP protein that cannot degrade KRP1 (Liu et al. 2016a). *KRP1* is up-regulated and KRP1 protein is stabilized in the *atprmt5* mutant. Since KRP1 negatively regulates cell division, cell division and shoot regeneration are inhibited in this mutant (Liu et al. 2016a).

### Histone acetylation-regulated plant regeneration

The level of histone acetylation is regulated by histone acetyltransferases (HATs) and histone deacetylases (HDACs) (Kumar et al. 2021). HATs activate gene expression by catalyzing the acetylation of histone lysine tails, while HDACs remove acetyl groups to repress gene expression. HATs and HDACs can add or remove acetylation at multiple lysine sites, including lysine 9 of histone H3 (H3K9), lysine 14 of histone H3 (H3K14), lysine 36 of histone H3 (H3K36), lysine 5 of histone H4 (H4K5), lysine 8 of histone H4 (H4K8), lysine 12 of histone H4 (H4K12), and lysine 16 of histone H4 (H4K16) (Kumar et al. 2021).

Histone acetylation levels change dynamically during various regeneration processes. During wound-induced callus formation, acetylation at lysine 9/14 of histone H3 (H3K9/14ac) and acetylation at lysine 27 of histone H3 (H3K27ac) accumulate on genes that are up-regulated by wounding, such as *WIND1*, *ERF113/RAP2.6L*, and *LBD* (Rymen et al. 2019). The histone acetyltransferases HISTONE ACETYLTRANSFERASE OF THE GNAT FAMILY 1 (HAG1) and HAG3 promote callus formation during wounding, as the callus formation rate is significantly reduced in *hag1* and *hag3* mutants (Rymen et al. 2019). HAG1 also promotes the transition of callus to shoots. During de novo shoot regeneration, HAG1 catalyzes the acetylation of *WOX5*, *SCR*, *PLT1*, and *PLT2* and promotes their expression, allowing the callus to acquire competence for shoot regeneration (Fig. 1B) (Kim et al. 2018). In addition to HATs, HDACs also affect plant regeneration. Inhibiting HDAC activity in *Arabidopsis* using the chemical inhibitor Trichostatin A (TSA) induced the transformation of hypocotyls into callus (Furuta et al. 2011) and induced somatic embryogenesis in the absence of auxin (Wójcikowska et al. 2018). Consistent with this observation, explants with a knocked-down expression of *HISTONE DEACETYLASE 19* (*had19*) showed enhanced embryogenic responses. Specifically, HDA19 inhibits somatic embryogenesis by negatively regulating *LEC1*, *LEC2*, and *BBM* expression by reducing their acetylation levels (Fig. 1B) (Morończyk et al. 2022). However, when leaves were used as the explant, TSA inhibited callus formation (Lee et al. 2016). Consistently, both *hda9* and *hd-tuins protein1* (*hdt1*) mutants show reduced callus induction from leaves (Lee et al. 2016).

In rice, mature embryos are used as explants for callus formation. TSA treatment inhibited the formation of rice callus (Zhang et al. 2020). OsHDA710 decreases the acetylation levels of the transcriptional repressor genes *OsARF18* and *OsARF22*, thereby activating *OsPLT1* and *OsPLT2* to promote callus formation (Fig. 1B) (Zhang et al. 2020). However, low concentrations of TSA promoted callus and shoot formation from mature wheat embryos, whereas high concentrations of TSA inhibited these processes (Bie et al. 2020). In addition, treatment with the histone deacetylase inhibitor sodium butyrate enhanced regeneration in wheat (Bie et al. 2020). Therefore, HDACs play various roles in the regeneration of different explants in different species. This variability is likely due to the promiscuous nature of histone acetylation modifiers, which modify multiple lysine residues of different histones.

### The roles of histone variants in plant regeneration

In addition to histone modification, histone variants affect chromatin status and gene transcription (Foroozani et al. 2021). For instance, the histone variant H2A.Z has dual functions in transcriptional activation and repression (Kumar 2018). In rice callus, H2A.Z is enriched at the 5′ ends of highly expressed genes, while inactive gene bodies are covered by H2A.Z (Zhang et al. 2017a). The atypical H3 variant HISTONE THREE RELATED 15 (H3.15) is involved in cell fate reprogramming during plant regeneration in Arabidopsis (Yan et al. 2020). H3.15 lacks the K27 residue that is trimethylated, so its replacement would dilute H3K27me3 levels (Yan et al. 2020). The H3.15-encoding gene *HISTONE THREE RELATED 15* (*HTR15*) is gradually up-regulated by auxin signaling during callus formation induced by wounding or culture on CIM (Yan et al. 2020). During callus formation, H3.15 is deposited on *WOX11* and helps remove H3K27me3, thus promoting *WOX11* expression and callus formation (Yan et al. 2020). Consistently, the *htr15* mutant has reduced callus formation ability (Yan et al. 2020).

## Chromatin accessibility dynamics are associated with the cell fate transition during plant regeneration

Chromatin accessibility dynamics are important for the regulation of gene expression and are in turn generally regulated by ATP-dependent chromatin remodeling complexes (CRCs). Remodelers can alter the accessibility of a specific genomic region to regulate DNA–histone interactions by changing the position, occupancy, and composition of nucleosomes using energy from ATP hydrolysis. Remodelers are highly conserved. Four subfamilies of remodeler complexes have been characterized in plants: CHROMODOMAIN HELICASE DNA BINDING (CHD), SWITCH DEFECTIVE/SUCROSE NON-FERMENTING (SWI/SNF), IMITATION SWITCH (ISWI), and INOSITOL REQUIRING 80/SWI2/SNF2-RELATED 1 (INO80/SWR1) (Han et al. 2015; Ojolo et al. 2018).

*Arabidopsis* INO80 and the histone chaperones NAP1-RELATED PROTEIN1 (NRP1) and NRP2 synergistically regulate inflorescence meristem (IM) size and RAM activity by affecting the expression of auxin-related genes and preventing DNA damage to maintain chromatin stability (Kang et al. 2019). *PICKLE* (*PKL*) is a *CHD3* homolog in *Arabidopsis* that facilitates root meristem activity (Aichinger et al. 2011) and maintains root cell identity to limit embryogenesis by regulating the expression of the PRC2-encoding genes *CLF* and *SWN* (Aichinger et al. 2009). BRAHMA (BRM) is an SWI/SNF chromatin remodeling ATPase that maintains root stem cell activity by directly targeting *PIN* genes (Yang et al. 2015). SPLAYED (SYD) is a SWI2/SNF2-like protein in the SNF2 subclass whose eviction, combined with the deposition of H3K27me3 at the *WUS* promoter, contributes to terminate floral meristem development in *Arabidopsis* (Sun et al. 2019)*.* Thus, chromatin remodelers generally participate in the regulation of meristem identity in plant tissues. However, few reports have documented how manipulating chromatin remodelers alters chromatin accessibility to influence plant regeneration. In monocot wheat, the regeneration efficiency is generally low (Wang et al. 2017; Zhang et al. 2018b). Co-expressing *GROWTH REGULATING FACTOR* and *4-GRF INTERACTING FACTOR 1* (*TaGRF4-TaGIF1*) greatly promoted regeneration in different wheat varieties (Debernardi et al. 2020). GIF recruits SWI/SNF chromatin remodeling complexes to its target genes to open the chromatin structure, thus allowing GRF4 to regulate downstream gene expression (Kim 2019; Luo and Palmgren 2021). Moreover, GIF1 functions together with GRFs to recruit SWI/SNF chromatin remodeling complexes to shape inflorescence architecture in maize (*Zea mays*) (Li et al. 2022).

In *Arabidopsis*, auxin treatment altered the chromatin accessibility of genes related to meristems and the cell cycle, such as *CYCLIN DEPENDENT KINASE B2;1* (*CDKB2;1*) and *PLT7*, to rewire the cell totipotency network and drive somatic embryogenesis (Wang et al. 2020)*.* A comparison of immature embryos and seedling explants revealed that open chromatin and the activated expression of embryonic genes such as *ABA INSENSITIVE 3* (*ABI3*), *BBM*, *FUSCA 3* (*FUS3*), *LEC1*, and *LEC2* are required for somatic embryogenesis in *Arabidopsis* (Wang et al. 2020). Similarly, in wheat, the gain of chromatin accessibility, along with the activation of key genes (such as *TaBBM* and *TaWOX5*) that mediate the cell fate transition, occurs during callus induction from immature embryos (Liu et al. 2022b). During shoot regeneration from pluripotent callus in *Arabidopsis*, root identity genes such as *WOX5* gradually lose their chromatin accessibility, while shoot identity genes such as *PHYTOCHROME INTERACTING FACTOR 1* (*PIF1*) gain chromatin accessibility. Furthermore, the chromatin states of genes related to epidermal cell differentiation, CK responses, and secondary metabolism gradually become more open (Wu et al. 2022). In rice, chromatin is generally more open in the callus than in seedlings, with 58% more DNase I hypersensitive sites in the callus that are positively correlated with transcription (Zhang et al. 2012). During the process of protoplast generation from leaf mesophyll cells in *Arabidopsis*, more accessible chromatin regions are created, leading to the random activation of *WUS*, which ultimately promotes regeneration (Xu et al. 2021).

Therefore, an accessible chromatin environment leads to higher totipotency, which is required for regeneration. Chromatin accessibility dynamics are associated with changes in the expression of key genes that drive the cell fate transition during different steps of plant regeneration. However, the general or specific roles of individual chromatin remodelers in plant regeneration remain unclear.

## DNA methylation status affects plant regeneration

In plants, DNA methylation occurs on cytosine, including symmetrical CG methylation, CHG methylation, and asymmetric CHH methylation (Law and Jacobsen 2010). The establishment, maintenance, and removal of DNA methylation marks are catalyzed by different enzymes (Law and Jacobsen 2010). DOMAINS REARRANGED METHYLTRANSFERASE 2 (DRM2) catalyzes de novo methylation, whereas the maintenance of DNA methylation requires different enzymes: CG methylation is maintained by DNA METHYLATRANSFERASE 1 (MET1, also known as DMT1), CHG methylation is maintained by CHROMOMETHYLASE 3 (CMT3), and CHH methylation is maintained by DRM2 and CMT2 (Zhong et al. 2014). DNA demethylation is initially mediated by DNA glycosidases, including DEMETER (DME), REPRESSOR OF SILENCING 1 (ROS1), DEMETER-LIKE 2 (DML2), and DML3 (Law and Jacobsen 2010).

Significant changes in DNA methylation levels both globally and at local key genes occur during multiple plant regeneration processes. Compared to leaves, global CHG methylation levels are higher and CHH methylation levels are lower in callus, which is consistent with the up-regulation of *CMT3* and down-regulation of *CMT2* in callus (Shim et al. 2022). Cell proliferation-related genes, including *PLT1*, *PLT2*, *ORIGIN RECOGNITION COMPLEX 1* (*ORC1*), *REPLICATION FACTORC 2* (*RFC2*), *MITOTIC ARREST DEFICIENT 1* (*MAD1*), and *DISRUPTION OF MEIOTIC CONTROL 1* (*DMC1*), are hypomethylated in callus (Shim et al. 2022). The binding motifs of the circadian rhythm regulator genes *CIRCADIAN CLOCK-ASSOCIATED 1* (*CCA1*) and *LATE ELONGATED HYPOCOTYL* (*LHY*) are enriched in these CHH-hypomethylated regions (Shim et al. 2022). Indeed, CCA1 directly binds to the promoter of the cell division-related gene *ORC1* to inhibit its expression, which may be related to the high CHH methylation levels of this promoter in leaves (Shim et al. 2022). During callus formation, CCA1 is inhibited and the CHH methylation level of the *ORC1* promoter decreases, thus releasing the expression of *ORC1* and enhancing cell proliferation (Shim et al. 2022).

DNA methyltransferases affect shoot regeneration. Compared to wild-type *Arabidopsis*, both the *met1* single mutant and *drm1 drm2 cmt3* (*ddc*) triple mutant show enhanced competence for shoot regeneration (Shemer et al. 2015; Liu et al. 2018a; Shim et al. 2021). Furthermore, the *ddc* mutant regenerated shoots directly from roots on SIM without inducing callus formation (Shemer et al. 2015). During the two-step shoot regeneration process, *MET1* is highly expressed in the callus under the activation of ATE2FA (E2FA), and its expression is down-regulated on SIM (Liu et al. 2018a). MET1 maintains the DNA methylation of *WUS* and inhibits *WUS* expression in the callus (Fig. 1C) (Liu et al. 2018a). When *MET1* was mutated, *WUS*, the CK signaling genes *ARR1* and *ARR10*, and the blue light receptor gene *CRYPTOCHROME 1* (*CRY1*) were activated to promote shoot regeneration (Liu et al. 2018a; Shim et al. 2021). Similarly, the up-regulation of *WUS* in the *ddc* mutant resulted in the direct conversion of roots into shoots on SIM (Fig. 1C) (Shemer et al. 2015). However, treatment with 5-azacytidine, which inhibits DNA methylation, has different effects on regeneration in different species. 5-azacytidine promoted the transformation of peach leaves to callus (Zheng et al. 2022) but inhibited callus formation in strawberry (*Fragaria ananassa*) (Liu et al. 2022a). In addition, treatment with 5-azacytidine enhanced somatic embryogenesis in Arabidopsis (Grzybkowska et al. 2018) but inhibited this process in rice (Hsu et al. 2018). These findings suggest that DNA methylation plays diverse roles in the regeneration of different plant species.

Plant regeneration competence is affected by the explant’s age and variety, which are also related to DNA methylation. The regeneration capacity of *Boea hygrometrica* leaves decreases during aging, which may be related to the high CHH methylation levels in mature leaves (Sun et al. 2020). There are significant differences in somatic embryogenesis competence between the cotton (*Gossypium hirsutum*) cultivars Yuzao1 and Lumian1, which may be related to the level of CHH methylation (Guo et al. 2020). Yuzao1 has a high somatic embryo induction rate and CHH hypomethylation, whereas Lumian1 has a low somatic embryo induction rate and CHH hypermethylation (Guo et al. 2020). Therefore, high DNA methylation levels reduce the regeneration ability of plants.

## MicroRNA levels are associated with plant regeneration capacity

miRNAs are a class of 21-nt non-coding small RNAs that reduce gene transcription by targeting mature mRNAs (Axtell 2013). miRNAs such as miR156, miR160, miR167, miR319, and miR393 are involved in plant regeneration via the direct or indirect regulation of auxin and CK signaling genes.

miR156 is involved in several age-related developmental processes (Xu et al. 2016; Guo et al. 2017). The shoot regeneration capacity of *Arabidopsis* and tobacco decreases with plant age, which can be compensated for by overexpressing *MiR156* (Zhang et al. 2015). SQUAMOSA PROMOTER BINDING PROTEIN-LIKE 9 (SPL9), encoded by a gene targeted by miR156, directly binds to type-B ARR genes, including *ARR1*, *ARR2*, *ARR10*, and *ARR12*, to impair CK responses (Fig. 1D) (Zhang et al. 2015). High levels of miR156 inhibited *SPL9* expression at the juvenile stage of *Arabidopsis* seedlings (Zhang et al. 2015). After juvenile-to-adult transition, decreased miR156 levels led to the up-regulation of *SPL9* and the inhibition of CK responses, thus weakening the capacity for shoot regeneration (Zhang et al. 2015). The miR156-SPL regulatory circuit plays a similar role in somatic embryogenesis in citrus. For the majority of citrus cultivars, the callus gradually loses its embryogenesis capacity and fails to differentiate into shoots after long-term culture (Long et al. 2018). miR156 levels are significantly lower in non-embryonic than in embryonic callus, while its target genes *CsSPL3* and *CsSPL14* show the opposite trend (Long et al. 2018). The expression levels of *CsSPL3* and *CsSPL14* are highly negatively correlated with somatic embryogenesis capacity in different citrus varieties (Long et al. 2018). In the orange varieties ‘Anliu’, ‘Newhall’, ‘Valencia’, and ‘American’ sour orange, which can undergo somatic embryogenesis, the expression levels of *CsSPL3* and *CsSPL14* are relatively low, while in varieties with weak competence for somatic embryogenesis, the expression levels of *CsSPL3* and *CsSPL14* are high (Long et al. 2018). These observations suggest that miR156-SPLs are involved in regulating age-dependent and variety-specific somatic embryogenesis in citrus.

Similar to miR156, miR319 also promotes shoot regeneration by affecting CK responses. The target genes of miR319 are *TCP3* and *TCP4*, encoding proteins that directly activate *ARR16*, which encodes a negative regulator of shoot regeneration (Fig. 1D) (Yang et al. 2020). Loss-of-function of HUA ENHANCER 1 (HEN1), a small RNA methyltransferase that stabilizes miR319, decreased miR319 levels, leading to the up-regulation of *TCP3* and *TCP4*, which in turn activated *ARR16* and inhibited shoot regeneration (Yang et al. 2020).

miRNAs also affect auxin signaling during plant regeneration. miR160 targets *ARF10* and inhibits auxin signaling, which in turn inhibits callus and shoot regeneration (Fig. 1D) (Qiao et al. 2012; Liu et al. 2016b). Furthermore, the transcriptional repressor ARF10 binds directly to AuxRE in the promoter region of *ARR15*, which encodes a negative regulator of callus formation (Fig. 1D) (Liu et al. 2016b). Therefore, miR160 inhibits regeneration by affecting both auxin and CK signaling pathways. In cotton, miR167 negatively regulates somatic embryogenesis by targeting *ARF6* and *ARF8* (Fig. 1D) (Arora et al. 2020). In plants overexpressing the miR167 target mimic (*35S::MIM167*), *ARF6*, *ARF8*, the auxin-responsive gene *GRETCHEN HAGEN 3* (*GH3*), and the auxin transporter genes *AUXIN RESISTANT 1 (AUX1)*, *LIKE AUX1 3* (*LAX3)*, *PIN1*, and *PIN2* were significantly up-regulated, suggesting that miR167 promotes somatic embryogenesis by enhancing auxin signaling (Arora et al. 2020). In addition to affecting *ARF* expression, miRNAs also affect the expression of auxin receptor-encoding genes, such as *TRANSPORT INHIBITOR RESPONSE 1* (*TIR1)* and *AUXIN SIGNALING F-BOX 3* (*AFB3)*. *TIR1* and *AFB3* were up-regulated in a *miR393* mutant, and the capacity for shoot regeneration and somatic embryogenesis was higher in the mutant than in the wild type (Fig. 1D) (Wójcik and Gaj 2016; Wang et al. 2018).

## Conclusions and future prospects

Cell totipotency and cell fate determination are fundamental research topics in biology. Plant regeneration provides an excellent system for studying these topics. Multi-step cell fate transitions occur during plant regeneration, which are accompanied by chromatin landscape remodeling and transcriptome reprogramming, particularly for cell identity genes such as *WOX11*, *WOX5*, and *WUS*. Recent studies have improved our understanding of the functions of various epigenetic regulators, such as histone modification ‘writers’ and ‘erasers’, chromatin remodelers, DNA methyltransferases, and miRNAs, in shaping plant regeneration by altering the expression of cell identity genes. However, many open questions remain.

Cell identity is associated with the accessibility of specific portions of the genome, which is controlled by interactions between genomic DNA and nucleosomes containing various histones (Chen and Dent 2014). Altering DNA–histone interactions via chromatin modifiers would affect the transcriptional competency of genes associated with specific regions of the genome (Klemm et al. 2019). Since cell identity frequently switches during plant regeneration, multiple chromatin modifiers are required to broadly alter the accessibility of certain portions of the genome and specifically fine-tune the expression of key genes in coordination with the activity of specific TFs. One challenging question is how different chromatin modifiers function cooperatively to control regeneration. The specific expression or induction patterns of chromatin modifiers and their recruiters might differ for different targets or for the same targets but at different stages of regeneration. For example, the methyltransferase ATXR2 of H3K36me3 and demethylase JMJ30 of H3K9me3 both regulate *LBD16* and *LBD19*, but their regulation is interdependent (Lee et al. 2018a). However, different chromatin modifiers might function together at the same loci. For instance, the removal of H3K27me3 and gain of chromatin accessibility as well as increases in H3K4me3 at specific gene clusters were detected during the early callus induction step of wheat shoot regeneration from immature embryos (Liu et al. 2022b). The detailed mechanism that coordinates the activities of different chromatin modifiers remains to be elucidated.

Phytohormone signals, especially auxin and CK signals, are essential during plant regeneration. Auxin and CK signals are transmitted to downstream target genes via ARF (Powers and Strader 2020) and type-B ARR (Li et al. 2021) TFs, respectively. On the one hand, epigenetic regulators can directly affect the expression of *ARF* and *ARR* (Zhang et al. 2020) or targets of ARF and ARR by “hijacking” ARF and ARR (Lee et al. 2017, 2021) to deposit specific histone modifications that alter their transcriptional activity. On the other hand, certain epigenetic regulators are induced by auxin or CK signaling, showing specific expression patterns during regeneration (Lee et al. 2018b, 2021). Therefore, additional studies are needed to explore the relationship between plant hormonal signals and epigenetic regulators, particularly to establish how auxin and CK influence epigenetic regulators for global chromatin remodeling and thus the cell fate transition.

The mechanisms of epigenetic regulation of plant regeneration, such as chromatin accessibility, H3K27me3, and H3K4me3, are generally conserved among different species, indicating that knowledge obtained studying model plants can be transferred to less-studied species, such as crops with large and complex genomes [e.g., wheat, maize, and barley (*H*o*rdeum vulgare*)]. However, epigenetic regulators show diverse, pleiotropic effects; the same factor may behave differently or even in an opposite manner during different stages of the regeneration process. Moreover, orthologous factors might exhibit various functions during the same stage of regeneration in different species or even in different explants of the same species. For these pleiotropic effects, in addition to epigenetic regulators per se, more attention needs to be paid to stage-specific recruiters that set the ‘specificity’ of epigenetically modified targets. The diverse effects of orthologous factors are likely related to the different pre-existing cell identities in various explants or similar explants of different species. Special attention should be paid to characterizing the explant- or species-specific reprogramming of epigenomics during regeneration.

Regeneration is widely used during the production of genetically manipulated plants for agriculture. Whereas in *Arabidopsis*, transgenic or genome-edited plants can be directly generated using the floral dip method (Clough and Bent 1998), major crops, including rice, wheat, and maize, require long-term tissue culture (Hiei et al. 2014). The efficiency of genetic transformation methods of crops has been improved by optimizing their regeneration systems (Hayta et al. 2019) and by the ectopic expression of genes encoding key regeneration factors such as WUS, BBM, and WOX5 (Lowe et al. 2016; Wang et al. 2022). However, due to the diversity among species and explants, not all factors that function in *Arabidopsis* regeneration can improve the efficiency of the genetic transformation of crops. Therefore, it is important to systematically study the regeneration processes of crops and to identify ‘novel’ factors that can enhance the efficiency of crop regeneration. Several recent studies have systematically analyzed gene expression and chromatin dynamics during the regeneration process of rice (Zhao et al. 2020; Shim et al. 2020), wheat (Liu et al. 2022b), and barley (Suo et al. 2021), providing valuable resources for mining key factors that enhance regeneration, such as TaDOF3.4 and TaDOF5.6 in wheat (Liu et al. 2022b). However, more in-depth analysis is still urgently needed to better understand the regeneration process and improve the genetic transformation efficiency of crops.

Finally, the development of single-cell and spatial omics technologies (Xia et al. 2022) provides additional tools for tracing cells with regenerative origins in various explants and exploring the heterogeneity of callus in the same generation or during transmission to the next generation (Mironova and Xu 2019; Xu et al. 2021; Zhai and Xu 2021). Such analyses will further enhance our mechanistic understanding of plant regeneration, thereby facilitating the development of advanced crop breeding tools.

## Data Availability

All data generated or analyzed during this study are included in this published article and its supplementary information files.
